# CCL4 enhances preosteoclast migration and its receptor CCR5 downregulation by RANKL promotes osteoclastogenesis

**DOI:** 10.1038/s41419-018-0562-5

**Published:** 2018-05-02

**Authors:** Dabin Lee, Kyung-Ju Shin, Dong Wook Kim, Kyung-Ae Yoon, Young-Jin Choi, Bom Nae Rin Lee, Je-Yoel Cho

**Affiliations:** Department of Biochemistry, BK21 PLUS Program for Creative Veterinary Science Research and Research Institute for Veterinary Science, College of Veterinary Medicine, Seoul National University, Seoul, South Korea

## Abstract

Chemokine CCL4 (MIP-1β) is released from osteoblast cells to restore the homeostasis of hematopoietic stem cells during the activation of bone marrow. In this study, we investigated the function of CCL4 and its receptor CCR5 during osteoclastogenesis. CCL4 promoted the migration and viability of preosteoclast cells. However, CCL4 had no direct effect on the receptor activator of nuclear factor-κB ligand (RANKL)-induced osteoclast differentiation in mouse preosteoclast cells. In addition, CCR5 expression was rapidly reduced by RANKL treatment, which was recovered by IFN-γ during osteoclastogenesis. CCR5 downregulation by RANKL was mediated by MEK and JNK in preosteoclast cells and promoted osteoclastogenesis. These results suggest that CCL4 can enhance the recruitment of preosteoclasts to bone in the early stage, and the reduction of CCR5 promotes osteoclastogenesis when RANKL is prevalent.

## Introduction

The stem cell niche is a microenvironment where stem cells interact with themselves, other cells, secreted factors, immunological factors, the extracellular matrix, and physical factors^1^. Above all, the hematopoietic stem cell niche is important in hematopoiesis, inflammation, and bone homeostasis. Hematopoietic stem cells (HSC) are precursor cells of blood and immune cells, including monocytes/macrophages that arise from osteoclast progenitor cells^2^. There are two forms of hematopoietic stem cell niche: one is an endosteal niche (osteoblastic niche), and the other is a sinusoidal vascular niche. Among them, the osteoblastic niche contributes to the maintenance of the HSC balance, quiescence, and activation^3^.

Bone marrow is a niche where HSCs are maintained and hematopoiesis and osteoclastogenesis occur. Bone marrow is suppressed by diverse sources of stress conditions, such as oxidation, anemia, hypoxia, radiation, cytotoxic chemotherapy, and inflammation^4–7^. Suppression of the bone marrow leads to a reduction in the function of bone marrow, including osteoclastogenesis. At that time, various inflammatory factors, such as cytokines and chemokines, are secreted from the diverse niche cells^8,9^.

Previous studies in our lab have investigated the expression of genes encoding secreted proteins that enforce HSC niche restoration after 5-FU treatment. After administration of a sublethal dose of 5-FU, HSC niche-related gene expression in osterix (Osx)-positive osteoprogenitor cells isolated from Osx-GFP::Cre transgenic mouse femurs and tibiae was examined by RNA-seq analysis. Among the regulated genes, the genes encoding secreted proteins and cellular membrane proteins were further analyzed. Genes that were shown to be upregulated in 5-FU-activated Osx-positive cells in the RNA-seq analysis were selected (Supplementary Table 1). To validate the expression of these candidate genes from the RNA-seq analysis in 5-FU-activated Osx-positive cells, real-time RT-PCR was performed (Supplementary Fig. 1), and the results revealed that the expression of CCL4 was dramatically increased in activated bone marrow niche cells in the 5-FU treatment group. Based on these previous results, CCL4 was chosen as a reinforcing factor of endosteal niche function.

CCL4, also known as macrophage inflammatory protein-1β (MIP-1β), is known to play a role in chemotactic activity in the immune cells^10^. CCL4 can activate chemokine receptor CCR5, which is involved in diverse immune responses^11^. Among the diverse chemokines, CCL4 and its receptor CCR5 have been a focus of research on HIV^12–15^. CCL4 is also secreted by CD8 + T cells^16,17^ and attracts monocytes, natural killer cells, and other immune cells^18^. CCR5 is mainly expressed on T cells, macrophages, dendritic cells, eosinophils, and microglia^19^. In addition, CCR5 has been demonstrated to be involved in cancer^20^. For example, breast cancer metastasis is promoted by CCL5–CCR5 activation^21^. The tumor suppressor miRNA-107 has also been reported to directly target CCR5 and antagonizes the proliferation and invasion of cervical cells^20^.

Osteoclasts are developed by the fusion of macrophage cells of hematopoietic lineage. Receptor activator of nuclear factor kappa-B ligand (RANKL) and macrophage colony-stimulating factor (M-CSF) from the osteoblast and stromal cells are essential for osteoclastogenesis^22^. The monocytes/macrophages stimulated by M-CSF and RANKL induce the expression of osteoclast-related genes such as cathepsin K (CTSK), tartrate-resistant acid phosphatase (TRAP), calcitonin receptor, and the β3-integrin. As a result, the mature osteoclasts play a role in bone resorption^23^. There are several chemokines, such as CCL9, CCL9, and CX3CL1, and their receptors that are related to osteoclastogenesis^24–26^, but the relationship between CCL4 and osteoclast differentiation is still unknown. Interestingly, several chemokine receptors, including CX3CR1 and CXCR6, are also known to be downregulated by RANKL^27,28^. Based on previous studies, the hypothesis that CCL4 and its receptor CCR5 inhibit osteoclast differentiation by RNAKL was established.

In this study, the functions of CCL4 and its receptor CCR5 were investigated in preosteoclast cells from mouse bone marrow to better understand the mechanisms of this chemokine and its receptor in osteoclastogenesis.

## Results

### CCL4 does not enhance RANKL-induced osteoclast differentiation

**Fig. 1:**
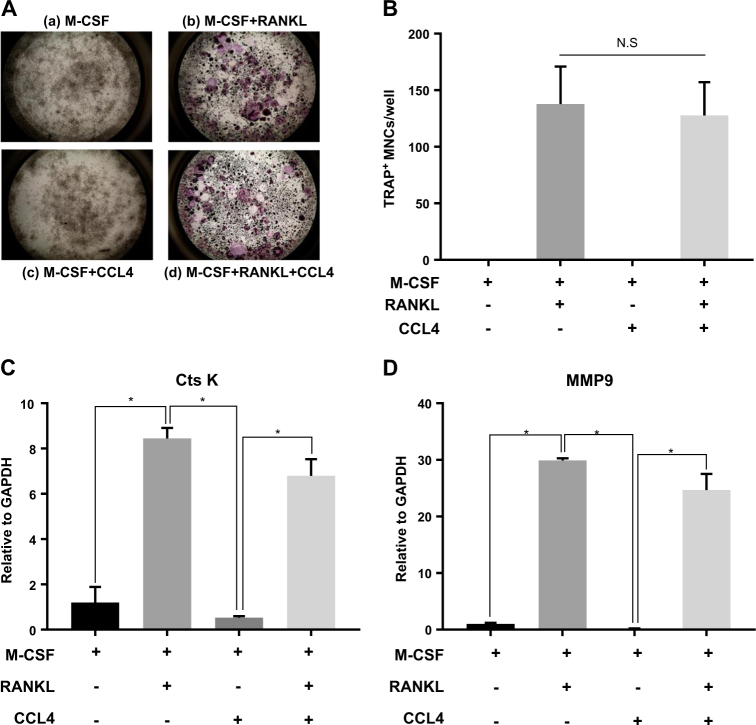
CCL4 does not augment RANKL-induced osteoclastogenesis. **a** Mouse monocytes were differentiated in the presence of recombinant (a) M-CSF (30 ng/ml), (b) M-CSF (30 ng/ml) + RANKL (50 ng/ml), (c) M-CSF (30 ng/ml) + CCL4 (10 ng/ml), or (d) M-CSF (30 ng/ml) + RANKL (50 ng/ml) + CCL4 (10 ng/ml) for 6 days. Osteoclast cells were induced by M-CSF + RANKL treatment, but the differentiated cells were not altered by the addition of CCL4 treatment. The cells were stained for TRAP. **b** TRAP-positive cells were counted as osteoclasts (≥3 nuclei). Gene-expression profiles related to osteoclasts were determined by real-time PCR. **c** Expression of cathepsin K; **d** expression of MMP9. The data are presented as the mean ± S.D. (*n* = 3; *P-value < 0.05, one-way ANOVA)

It was first investigated whether CCL4 had a direct effect on osteoclast differentiation. In the presence of M-CSF and RANKL, preosteoclast cells were well differentiated into osteoclasts (Fig. 1a–panel b), but CCL4 treatment (500 pg, 1, 5, and 10 ng) did not show any effect on differentiation (Fig. 1a-panel b, a–panel d, and Supplementary Fig. 3). TRAP staining showed that increased osteoclast cells by RANKL are not altered by CCL4 treatment (Fig. 1b). In contrast, high concentration of CCL4 treatment (25, 50, and 100 ng) induced cell death from 2 days after treatment (Supplementary Fig 4). Moreover, RANKL-mediated osteoclastogenesis is not blocked by CCL4 treatment, which is supported by the observation that RANKL-mediated induction of CtsK and MMP9 transcript is not inhibited by CCL4 treatment (Fig. 1c, d). These results suggested that CCL4 might not play a role in osteoclast differentiation process itself induced by M-CSF and RANKL. Additionally, we examined the expression of CCL4 during differentiation because of the possibility that CCL4 was induced and affected the differentiation when M-CSF and RANKL were treated by real-time PCR. As a result, M-CSF and RANKL were treated to induce differentiation (Supplementary Fig. 5C, D), but the expression of CCL4 was downregulated (Supplementary Fig. 5A). That is, CCL4 is not induced in osteoclast differentiation.

### CCL4 promotes the migration and viability of preosteoclast cells

**Fig. 2:**
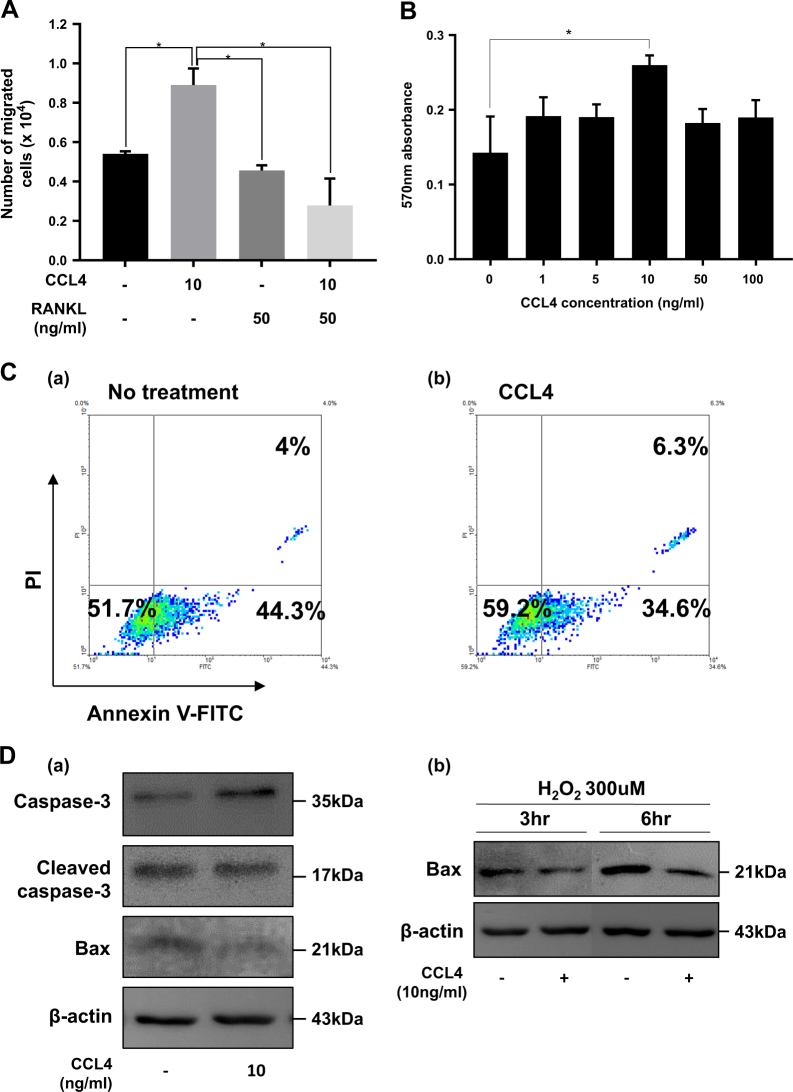
CCL4 promotes migration and viability of preosteoclast cells. **a** Migration assay. Preosteoclast cells were resuspended in serum-free cell culture media in each upper transwell chamber. After incubation for 2 h, cells that had migrated to the lower chamber were counted. The data are presented as the mean ± S.D. **b** Effects of CCL4 on the survival of preosteoclast cells *in vitro*. Cell viability was measured by MTT assay. Cells were treated with various concentrations (0–100 ng) of CCL4. The data are presented as the mean ± S.D. (*n* = 3; *P-value < 0.05, Student’s one-way ANOVA). **c** Preosteoclast cells were incubated in a medium with or without 10 ng/ml CCL4 for 3 days. To determine the apoptotic cell population, cells were stained with annexin V/PI staining and analyzed by flow cytometry (FACS) analysis. In each panel, the lower left quadrant (annexin V–/PI–) shows cells that are viable cell populations, the lower right (annexin V+/PI−) shows cells that are in the early stage of apoptosis, and the upper right (annexin V+/PI+) shows cells that are in the late stage of apoptosis. **d** Preosteoclast cells were incubated in a medium with or without 10 ng/ml CCL4 for 24 h. To confirm the anti-apoptotic effect of CCL4, we performed western blot assay with caspase-3, cleaved caspase-3, Bax, and β-actin antibodies

The chemotaxis of preosteoclast cells was investigated by a transwell migration assay to determine the function of CCL4 in preosteoclast cells. CCL4 induced preosteoclast cell migration when the lower chamber contained media including CCL4 (Fig. 2a). However, RANKL treatment resulted in an evident inhibition of cell chemotaxis mediated by CCL4 (Fig. 2a). The effect of CCL4 on the viability of the preosteoclast cells was measured by the MTT assay. Preosteoclast cells were treated with or without various concentrations of CCL4 for 3 days along with M-CSF (30 ng/ml). Compared to the cell viability exerted by M-CSF treatment alone, CCL4 treatment at a concentration of 10 ng/ml improved cell viability (Fig. 2b).

In addition to cell viability, CCL4 treatment showed the anti-apoptotic effect. FACS analysis with annexin V/PI staining showed that CCL4 treatment alone increased cell population that is negative for both annexin V/PI (Annexin V–/PI–) from 51.7 to 59.2%, and decreased early apoptotic cells (Annexin V+/PI−) from 44.3 to 34.6% (Fig. 2c–panels a, b). Additionally, western analysis with the cell lysates treated with CCL4 showed that total caspase-3 was increased and Bax was decreased, respectively, but no significant change was observed in cleaved caspase-3 (Fig. 2d–panel a). Moreover, CCL4 could decrease Bax expression level induced by H_2_O_2_ (Fig. 2d–panel b). These results revealed that CCL4 increased the viability of preosteoclast cells and protected the cells from apoptosis.

### RANKL downregulates CCR5 expression during osteoclastogenesis in preosteoclast cells

**Fig. 3:**
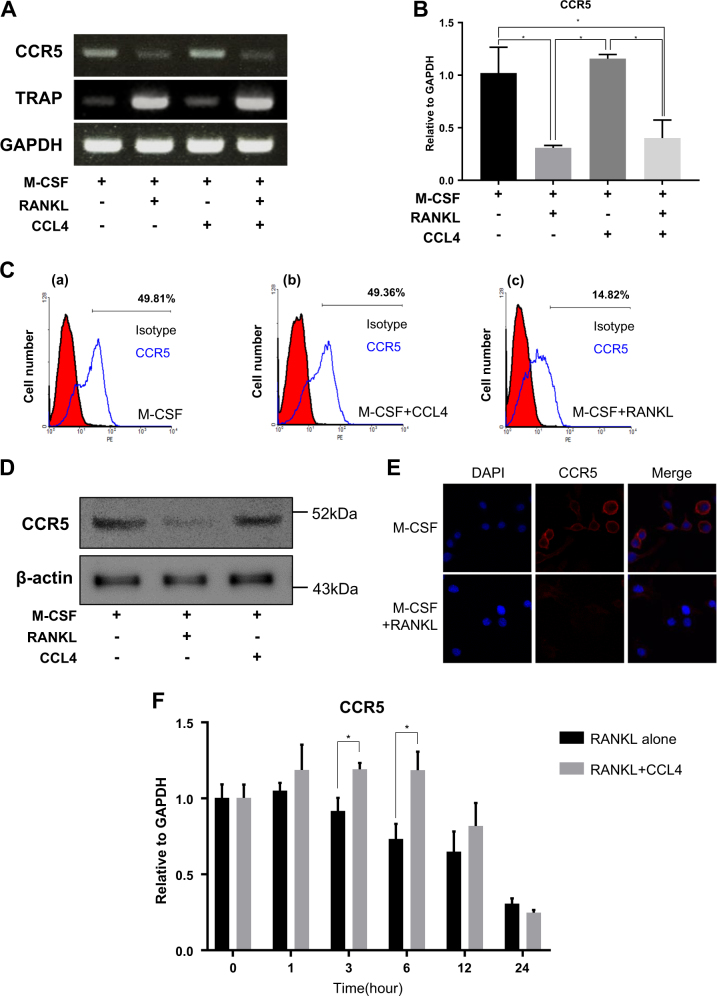
RANKL downregulated CCR5 expression in preosteoclast cells. **a** Preosteoclast cells were cultured for 4 days in the presence of recombinant M-CSF (30 ng/ml) with RANKL (50 ng/ml) and CCL4 (10 ng/ml). Total RNA was extracted and converted to cDNA, which was used for conventional RT-PCR and (**b**) real-time RT-PCR. **c** FACS analysis. Preosteoclast cells were pretreated with (a) M-CSF (30 ng/ml), (b) M-CSF (30 ng/ml) + CCL4 (10 ng/ml), or (c) M-CSF (30 ng/ml) + RANKL (50 ng/ml) for 48 h. **d** Western blot of CCR5 with treatment of RANKL or CCRL4-treated cell. **e** Immunofluorescence staining for nuclear (blue) and CCR5 (red) in macrophage cells. **f** Cells were stimulated with RANKL alone (50 ng/ml) or RANKL with CCL4 (10 ng/ml) for 0–24 h. The expression levels of CCR5 mRNA were measured by real-time RT-PCR and normalized to GAPDH. The data are presented as the mean ± S.D. (*n* = 3; *P-value < 0.05, two-way ANOVA)

During osteoclastogenesis, the expression levels of CCR5 mRNA were investigated in preosteoclast cells treated with or without RANKL (50 ng/ml) for 3 days. CCR5 expression was dramatically decreased with RNAKL treatment, regardless of the presence or absence of CCL4 (Fig. 3a–b). RANKL-induced reduction of CCR5 was also examined by flow cytometry (FACS) analysis, western blot, and immunofluorescence assays. These analyses showed that the number of cells expressing the CCR5 protein was significantly reduced by RANKL (Fig. 3c–e). To determine temporal profiles of CCR5 levels by RANKL, CCR5 mRNA levels were analyzed up to 24 h after RANKL treatment. The primary cells were treated with CCL4 and M-CSF to differentiate into macrophage lineages. CCR5 levels were downregulated from 3 h after RANKL treatment (Fig. 3f, black bar). When CCL4 was treated with RANKL (Fig. 3f, gray bar), the expression levels were maintained up to 6 h, but after 12 h, CCR5 levels were similarly downregulated compared to without CCL4 treatment. These results demonstrated that, even in the presence of CCL4, CCR5 downregulation occurred in parallel to RANKL-mediated osteoclastogenesis in preosteoclast cells.

### CCR5 inhibits osteoclast differentiation

**Fig. 4:**
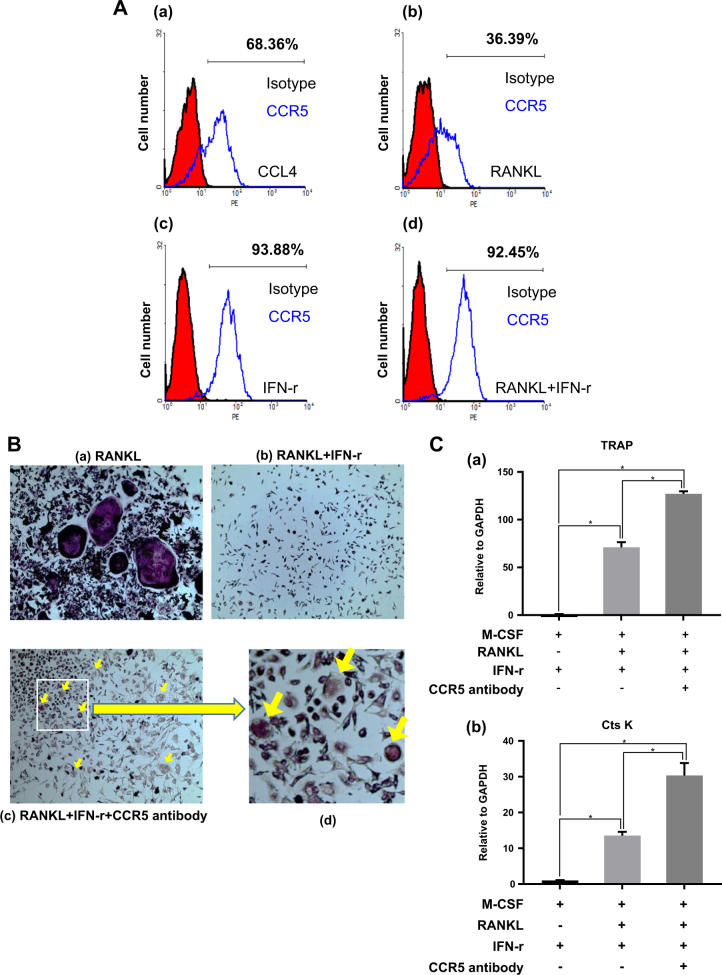
CCR5 inhibits differentiation to osteoclasts. **a** FACS analysis was done with CCR5 antibodies. For all groups, mouse monocytes were pretreated with 30 ng/ml M-CSF. The cells were treated with (a) CCL4 (10 ng/ml), (b) RANKL (50 ng/ml), (c) IFN-γ (100 U/ml), or (d) RANKL + IFN-γ for 48 h. **b** TRAP staining for the measure of osteoclast differentiation. Mouse monocytes were differentiated in the presence of recombinant 30 ng/ml M-CSF. The cells were treated with (a) RANKL (50 ng/ml), (b) RANKL + IFN-γ (100 U/ml), or (c) RANKL + IFN-γ + CCR5 antibody for 6 days. (d) is enlarged from (c). **c** The expression of (a) TRAP and (b) cathepsin K, osteoclast-related genes, was determined by real-time PCR. The data are presented as the mean ± S.D. (*n* = 3; *P-value < 0.05, one-way ANOVA)

To determine the biological meaning of CCR5 reduction during RANKL-mediated osteoclast differentiation, osteoclastogenesis was performed in the presence of IFN-γ. Previous reports demonstrated that IFN-γ, LPS, and H_2_O_2_ upregulate CCR5 expression in T-cells, monocytes, and other immune cells^29–32^. These factors are involved in osteoclast differentiation. In particular, IFN-γ is known to inhibit osteoclast differentiation^33^. Considering these studies, we hypothesized that CCR5 inhibits osteoclast differentiation. In order to prove our hypothesis that a high level of CCR5 inhibits osteoclast differentiation, osteoclast differentiation was investigated in the presence of IFN-γ. When the preosteoclast cells were treated with IFN-γ in the presence of RANKL, unlike with RANKL treatment alone (Fig. 4B–panel b), the cells were not differentiated into osteoblasts (Fig. 4b–panel b). IFN-γ induced 30% induction of CCR5 expression (68.36–93.88%), as compared with CCL4 treatment (Fig. 4a–panel a, c). It is notable that CCL4 treatment had no effect on the CCR5 expression 12 h after treatment (Fig. 3f). As shown again in Fig. 4A–panel b, treatment with RANKL dramatically decreased CCR5 levels. However, RANKL treatment did not affect CCR5 expression in the presence of IFN-γ (93.88–92.45%) (Fig. 4a–panels c, d). However, blocking CCR5 with its antibody in the presence of IFN-γ resulted in better osteoclast differentiation, although the differentiation was not fully recovered (Fig. 4b, panel c). This suggests that there might be other pathways for the inhibition of IFN-γ on osteoclast differentiation in addition to CCR5. However, our data suggest that CCR5 signal is involved in the inhibition of osteoclast differentiation. In addition to the TRAP staining (Fig. 4b), early osteoclastogenesis was also measured by the expression levels of osteoclast markers TRAP and cathepsin K. Both markers were significantly higher in the cells where CCR5 was blocked with its antibody in the presence of IFN-γ than in the cells where CCR5 was not blocked (Fig. 4c). Combined all together, these results suggest that CCR5 inhibits osteoclast differentiation.

### CCR5 reduction by RANKL was restored by blocking MEK and JNK signal

We then tested the signaling mechanism of the CCR5 downregulation by RANKL. Binding of RANKL to its receptor RANK induces several intracellular signaling molecules in preosteoclast cells, including MAPKs, Src kinase, and NF-κB^34^. To verify the signaling pathways causing the RANKL-induced reduction of CCR5 expression, the effects of various kinase inhibitors were examined, including a MEK inhibitor (U0126), p38 inhibitor (SB20380), JNK inhibitor (SP600125), NF-κB inhibitor (BAY 11-7082), and PI3K inhibitor (LY294002) (Supplementary Fig. 6). As shown in Supplementary Fig. 6A, treatment with 1–20 µM of the MEK inhibitor (U0126) restored the expression of CCR5 reduced by RANKL. In contrast, treatment with 5–30 µM of the p38 inhibitor (SB20380) did not affect RANKL-induced suppression of CCR5 (Supplementary Fig. 6B). Additionally, the JNK inhibitor, NF-κB inhibitor, and PI3K inhibitor slightly restored the levels of CCR5 (Supplementary Fig. 6C–E).

**Fig. 5:**
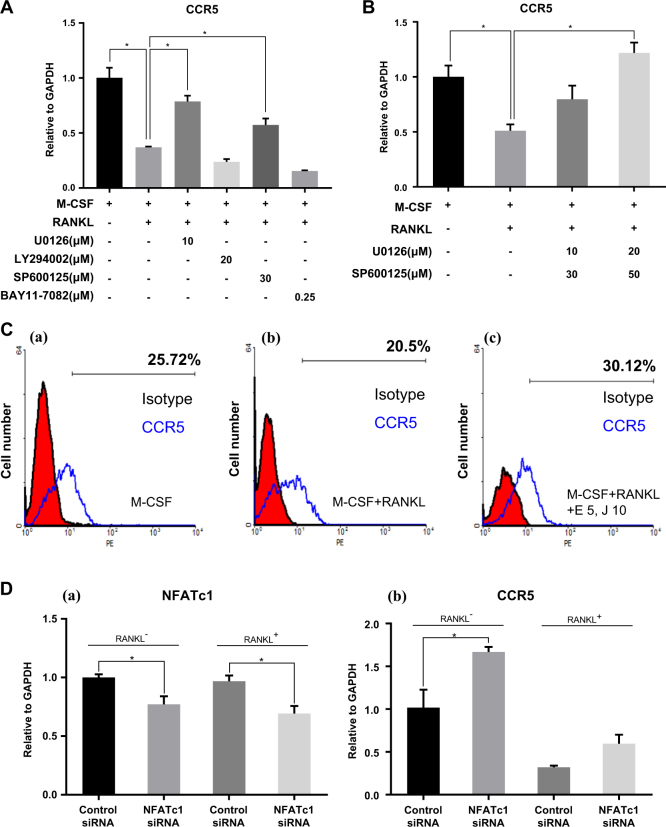
Inhibition of the RANKL-induced reduction of CCR5 mRNA. **a** Preosteoclast cells were treated with MEK inhibitor U0126 (10 µM), Akt inhibitor LY294002 (20 µM), JNK inhibitor SP600125 (30 µM), and NF-κB inhibitor BAY11-7082 (0.25 µM) as an indicated combination. Expression levels of CCR5 mRNA were measured by real-time RT-PCR and normalized to GAPDH. The data are presented as the mean ± S.D (*n* = 3; *P-value < 0.05, one-way ANOVA). **b** Preosteoclast cells were pretreated with U0126 (10 μM) + SP600125 (30 μM) or U0126 (20 μM) + SP600125 (50 μM). Expression levels of CCR5 mRNA were measured by real-time RT-PCR and normalized to GAPDH. The data are RANKL (50 ng/ml) stimulation for 24 h. Expression levels of CCR5 mRNA were measured by real-time RT-PCR and normalized to GAPDH. The data are presented as the mean ± S.D. (*n* = 3; *P-value < 0.05, one-way ANOVA). All the chemicals were dissolved in DMSO and used as a vehicle control in all experiments. **c** Expression levels of CCR5 were also detected by the anti-CCR5 antibody using flow-cytometry analysis. **d** Preosteoclast cells were transfected with NFATc1 siRNA or control siRNA, and the respective knockdown efficiency was analyzed by real-time RT-PCR with or without RANKL treatment. The expression level of (a) NFATc1 and (b) CCR5 (*n* = 2; *P-value < 0.05, one-way ANOVA)

Interestingly, when the MEK inhibitor was given with the JNK inhibitor, CCR5 levels were completely restored (Fig. 5b). To confirm these data, the cell surface CCR5 was measured by flow-cytometry analysis (Fig. 5c). The CCR5-positive cell proportion was again recovered by the MEK inhibitor plus JNK inhibitor (Fig. 5c–panel c). These results implied that RANKL-mediated downregulation of CCR5 expression was associated with the MEK and JNK signaling.

RANKL activates several intracellular signaling molecules such as MAPK, NF-kB, and NFATc1. It is also reported that RANKL activates NFATc1 through MEK/JNK signaling^35,36^. Therefore, we tested whether CCR5 downregulation by RANKL through MEK/JNK is also mediated by NFATc1. Knockdown of NFATc1 by siRNA on preosteoclast cells rescued CCR5 expression from the CCR5 downregulation by RANKL (Fig. 5d). Consequently, these data showed that the expression of CCR5 was also regulated by NFATc1, possibly through MEK/JNK pathway activated by RANKL.

## Discussion

Activation of chemokine expression has been known to occur in several stress conditions^37^. A previous RNA- sequencing analysis showed that CCL4 was significantly elevated in Osx-positive cells when acute bone marrow damage was induced by 5-FU treatment (Supplementary Table 1, Supplementary Fig. 1). In this study, the functions of CCL4 secreted from the osteoblastic niche and its receptor CCR5 were investigated in the effector preosteoclast cells.

First, osteoclast differentiation was performed to determine whether CCL4 promoted osteoclastogenesis. Osteoclasts are known to resorb bone tissue and release TGF-b, which recruits preosteoblast cells to remodel bone and enhances the overall osteoblastic niche condition^38^. However, our results revealed that CCL4 was not essential for osteoclast differentiation. TRAP staining showed that there was no significant difference in the differentiation of preosteoclast cells between cells treated with CCL4 and RANKL and cells only treated with RANKL (Fig. 1). This result indicated that CCL4 was not directly involved in the osteoclastogenetic process.

Hence, CCL4 functions in recruiting preosteoclast cells and improving the osteoblastic niche were investigated. In the migration assay, CCL4 promoted preosteoclast cell migration (Fig. 2). On the other hand, preosteoclast migration was reduced upon RANKL treatment. During osteoclastogenesis, the expression of the CCL4 receptor, CCR5, which plays a significant role in osteoclast differentiation, was repeatedly observed to be inhibited by RANKL (Fig. 3). These results demonstrated that RANKL inhibited cell migration stimulated by CCL4 (Fig. 2) through downregulation of its receptor CCR5 (Fig. 3a–e).

Another function of CCL4 was promoting cell viability. Some chemokines are known to improve cell viability and enhance the resistance to apoptosis^39^. Our data also showed that CCL4 enhanced cell viability (Fig. 2c) and prevented apoptosis in preosteoclast cells (Fig. 2d, e). These results suggest that CCL4 plays a role in recruiting preosteoclast cells in good condition during the early osteoclast differentiation process and induces differentiation into mature osteoclasts.

During osteoclastogenesis, RANKL decreases the expression of several chemokine receptors, and the signaling pathways that are involved in the downregulation of chemokine receptors by RANKL have been studied^27,28^. Our data showed that CCR5 was downregulated by RANKL (Fig. 3). Next, the mechanism of RANKL-mediated downregulation of CCR5 during osteoclast differentiation was investigated. Comparatively, little is known about the mechanism and physiological role of downregulation of chemokine receptors, in contrast to upregulation of their expression.

The inhibition of CCR5 during osteoclastogenesis further extended the idea that high levels of CCR5 would hinder osteoclast differentiation. The first thing we were curious about the CCR5 was that its expression level was downregulated by RANKL which induces osteoclast differentiation. So, we have a question as to whether CCR5 should be downregulated during osteoclastogenesis. The best way in understanding the role of CCR5 in osteoclast differentiation is overexpression of CCR5, but it is difficult to perform with primary cells. So, IFN-Ɣ was considered to be the most appropriate when looking for an alternative that could increase CCR5. IFN-γ has been known to induce the expression of chemokine receptors including CCR5 and CCR3^29^. Thus, osteoclast differentiation was carried out in the presence of IFN-γ to maintain high levels of CCR5. In the presence of IFN-γ, the CCR5 level was elevated (Fig. 4a–panel c), and osteoclastogenesis was shown to not occur in our results (Fig. 4b–panel b). In addition, blocking CCR5 increased the expression of TRAP and cathepsin K, both osteoclast markers, compared to that in the IFN-γ-only treatment group (Fig. 4c). Obviously, our data showed that the increase in CCR5 expression inhibited osteoclast formation. Blocking CCR5 may not have completely rescued osteoclast differentiation due to other chemokine receptors such as CCR3, which may be similarly upregulated by IFN-γ^29^ and downregulated by RANKL treatment^40^. In our experiment, only CCR5 was blocked by an antibody, and CCR3 would be maintained at a high level in the presence of IFN-γ; thus, the increased level of CCR3 might inhibit osteoclastogenesis.

The RANKL-mediated signals, MEK and JNK, were identified to be involved in downregulation of CCR5 expression. Binding of RANKL to RANK initiates downstream signaling pathways that are related to osteoclast differentiation. ERK, JNK, p38, PI3K/AKT, and IKK1/2, and transcription factors NFAT, AP-1, c-Fos, and NF-κB are involved in osteoclast differentiation^23,27^. Figure 5 shows that the RANKL-induced reduction of CCR5 expression was controlled via two downstream signals of RANKL, MEK and JNK. RANKL activates the nuclear factor of activated T-cells, cytoplasmic 1 (NFATc1) through MEK and JNK signaling^36^. NFATc1 is a master transcription factor of osteoclastogenesis known to regulate the transcription of CCR5 as it binds to the CCR5 promoter^41,42^. Interestingly, a similar mechanism was found in the transcriptional regulation of chemokine receptor CX3CR1; NFAT1 regulates the CX3CR1 promoter in natural killer cells^27^. Based on the results that suggest that CCR5 is downregulated by RANKL through activation of MEK and JNK, and NFATc1 is activated by RANKL through the MEK and JNK signals, NFATc1 might regulate CCR5 expression. Further studies are necessary to investigate the association between CCR5 expression and NFATc1 in osteoclast differentiation. RANKL-mediated chemokine reduction in osteoclast differentiation is important to study to obtain a better understanding of the osteoblastic niche.

In conclusion, CCL4 was shown to affect the osteoblastic niche, just as chemokines have been shown to play roles, such as recruiting progenitor cells and maintaining viability, in the osteoblastic niche^43^. Although the CCL4 chemokine did not directly promote later osteoclast differentiation, our study revealed that CCL4 recruited viable preosteoclast cells for osteoclastogenesis.

## Materials and methods

### Reagents

The PI3K inhibitor (LY294002) and MEK inhibitor (U-0126) were purchased from Enzo Life Sciences (Plymouth Meeting, PA). The NF-κB inhibitor (BAY11-7082) and p38 inhibitor (SB203580) were purchased from Calbiochem (Ellisville, MO). The phospho-p44/p42 MAPK (Erk1/2) antibody was purchased from Cell Signaling Technology (Cell Signaling, Danvers, MA). PE anti-mouse CD195 (CCR5) and PE Armenian hamster IgG isotype control were purchased from Biolegend (Biolegend, San Diego, CA). Mouse CCR5 antibody was purchased from R&D System (R&D System, Minneapolis, MN). Recombinant murine MIP-1β (CCL4) and recombinant murine IFN-γ were purchased from Peprotech (Peprotech, Rocky Hill, NJ). Recombinant mouse RANKL and recombinant mouse M-CSF were purchased from R&D System (R&D System, Minneapolis, MN).

### Isolation and osteoclast differentiation of murine bone marrow-derived monocytes

Mouse bone marrow cells were isolated by flushing femurs and tibias from 8- to 9-week-old BALB/c mice and incubated for 24 h in MEM-α modification (1×) with 10% FBS and 1% penicillin/streptomycin. After 24 h, only non-adherent cells were collected and used for differentiation^44^. To initiate differentiation, non-adherent cells were cultured in MEM-α modification with 30 ng/ml recombinant mouse M-CSF (R&D, MN, USA). Then, the mouse bone marrow cells were differentiated into macrophages. The macrophage cultures in MEM-α modification with 20 ng/ml recombinant mouse M-CSF and 50 ng/ml recombinant mouse RANKL (R&D, MN, USA) were treated daily for 3 days to generate osteoclasts^23^. RANKL-treated preosteoclast cells had a multinuclear cell phenotype, as determined by tartrate-resistant acid phosphatase (TRAP) staining (Sigma-Aldrich, MO, USA). Osteoclasts were counted as TRAP-positive cells under light microscopy.

### MTT (3-(4,5-dimethylthiazol-2-yl)-2,5-diphenyltetrazolium bromide) assay

The MTT assay was done as an indicator of cell survival and/or growth. The assay determines the presence of live cells with functional mitochondria. Cell viability was measured by the 3-(4,5-dimethylthiazol-2-yl)-2,5-diphenyltetrazolium bromide (MTT) assay to determine relative cell growth^45^. Preosteoclast cells were placed into 48-well plates that were treated with varying concentrations (0, 1, 5, 10, 50, and 100 ng/ml) of CCL4 for 24 h. MTT (AMRESCO, Solon, OH) was added to each well and incubated for an additional 4 h at 37 °C. Following removal of the culture medium, the remaining crystals were dissolved in 200 μl of DMSO, and absorbance at 570 nm was measured.

### Annexin V/PI staining

For assays measuring apoptosis, the preosteoclast cells were cultured at a concentration of 3 × 10^6^ cells in a 60-mm Petri dish and treated as described. For measuring apoptosis, cells were harvested and stained with 5 µl of FITC Annexin V and 1 µl of the 100 µg/ml PI working solution for 15 min at room temperature. After the incubation period, 400 µl of 1 × annexin-binding buffer was added, and the samples were mixed gently and maintained on ice before analysis with flow cytometry.

### Migration assay

Monocyte chemotactic activity was tested using 5.0-μm-pore polycarbonate membrane inserted into transwell cell culture chambers (Corning, USA). Monocytes were washed with FBS-free medium and resuspended in 0.1% BSA medium at a density of 1 × 10^6^ cells per well in 48-well plates. Two-hundred microliters of aliquots of cell suspension were added to the upper compartment, while the lower compartments contained 600 µl of medium with or without 10 ng/ml CCL4 and with or without 50 ng/ml RANKL. After incubation for 2 h at 37 °C, cells that had migrated to the lower surface were counted.

### Reverse transcriptase-polymerase chain reaction (RT-PCR) and real-time RT-PCR analysis

Total RNAs from preosteoclast cells were isolated using TRIzol reagent (Ambion, Carlsbad, CA) according to the manufacturer’s recommendations. Then 500 ng to 1 µg of total RNA from each sample were reverse- transcribed with an Omniscript Reverse Transcription kit (QIAGEN, Hilden, Germany). The reverse transcription reaction was carried out at 37 °C for 1 h. To measure the expression of genes, fluorescence-based real-time RT-PCR was performed with the thermocycler (Bio-Rad, Hercules, CA). SYBR green and Go-Taq ® Flexi DNA polymerase (Promega, Madison, WI) were used for DNA amplification and detection. Relative mRNA expression levels were determined by △△Ct calculation^46^.

### Western blot analysis

Preosteoclast cells were incubated with M-CSF, RANKL, and CCL4. Then, whole-cell lysates were prepared in a RIPA buffer (25 mM Tris•HCl, pH 7.6, 150 mM NaCl, 1% NP-40, 1% sodium deoxycholate, and 0.1% SDS) (Thermo Scientific, Bellefonte, PA) containing a protease inhibitor cocktail (Roche, Mannheim, Germany). Lysates were incubated in ice for 30 min and centrifuged at 14,000 r.p.m. for 20 min. Protein concentrations were determined using the Bradford protein assay kit (Bio-Rad, Hercules, CA, USA). Equal amounts of cell-lysate protein (30 µg/lane) were subjected to SDS-PAGE and transferred to polyvinylidene difluoride (PVDF) membranes. Membranes were blocked with 5% BSA in 0.05% Tween 20 in Tris-buffered saline (TBST). The membranes were incubated with a CD195 (CCR5) antibody (R&D System, Minneapolis, MN) overnight at 4 °C. The membrane was washed three times with TBST and then incubated with the appropriate horseradish peroxidase-conjugated secondary antibodies for 1 h at room temperature. The blots were developed using a chemiluminescence detection system (ATTO Corporation, Tokyo, Japan) and exposed to an X-ray film.

### Flow cytometry

The expression of CCR5 on the surface of monocytes was assessed with PE anti-mouse CD195 (CCR5) (Biolegend, San Diego, CA). In brief, 3 × 10^5^ treated cells were washed twice with PBS, labeled for 30 min at 4 °C with PE anti-mouse CD195 (CCR5), and then washed twice with cold PBS. Finally, cells were washed and resuspended in PBS containing 3% FBS before flow-cytometry analysis with a FACS-scan flow cytometer (Becton-Dickinson, San Jose, CA).

### NFATc1 siRNA transfection

The mouse NFATc1 siRNA was purchased from Santa Cruz Biotechnology (Paso Robles, CA). Mouse macrophage cells were seeded into each well of six-well culture plates using MEM-α modification (1×) with 10% FBS without antibiotics. The next day, cells were transfected with Lipofectamine 3000 (Thermo Fisher Scientific, Carlsbad, CA) according to the manufacturer’s protocol. After 24–72 h, total RNA was isolated and confirmed by real-time PCR.

### Statistical analyses

Data are presented as the mean ± S.D. GraphPad Prism software (version 7.01, GraphPad software, Inc., CA, USA) was used for statistical analyses. Comparisons were performed using a Student’s *t*-test or one-way ANOVA followed by the Tukey–Kramer multiple-comparison tests. The cell survival data were analyzed by two-way ANOVA followed by Sidak multiple-comparison tests.

## Electronic supplementary material

Supplementary Table 1

Supplementary Figure 1, Supplementary Figure 2, Supplementary Figure 3, Supplementary Figure 4, Supplementary Figure 5, Supplementary Figure 6, Supplementary Figure 7, Supplementary Figure 8

Supplementary figure legends
