# Supplementary material for: CCL4 enhances preosteoclast migration and its receptor CCR5 downregulation by RANKL promotes osteoclastogenesis
Source: Cell Death Dis. 2018 May 2;9(5):495. doi: 10.1038/s41419-018-0562-5 (PMC5931580; doi:10.1038/s41419-018-0562-5)
Supplement: Supplementary file 2 — Supplementary Figure 1, Supplementary Figure 2, Supplementary Figure 3, Supplementary Figure 4, Supplementary Figure 5, Supplementary Figure 6, Supplementary Figure 7, Supplementary Figure 8 [file 41419_2018_562_MOESM2_ESM.pptx]

## Slide 1
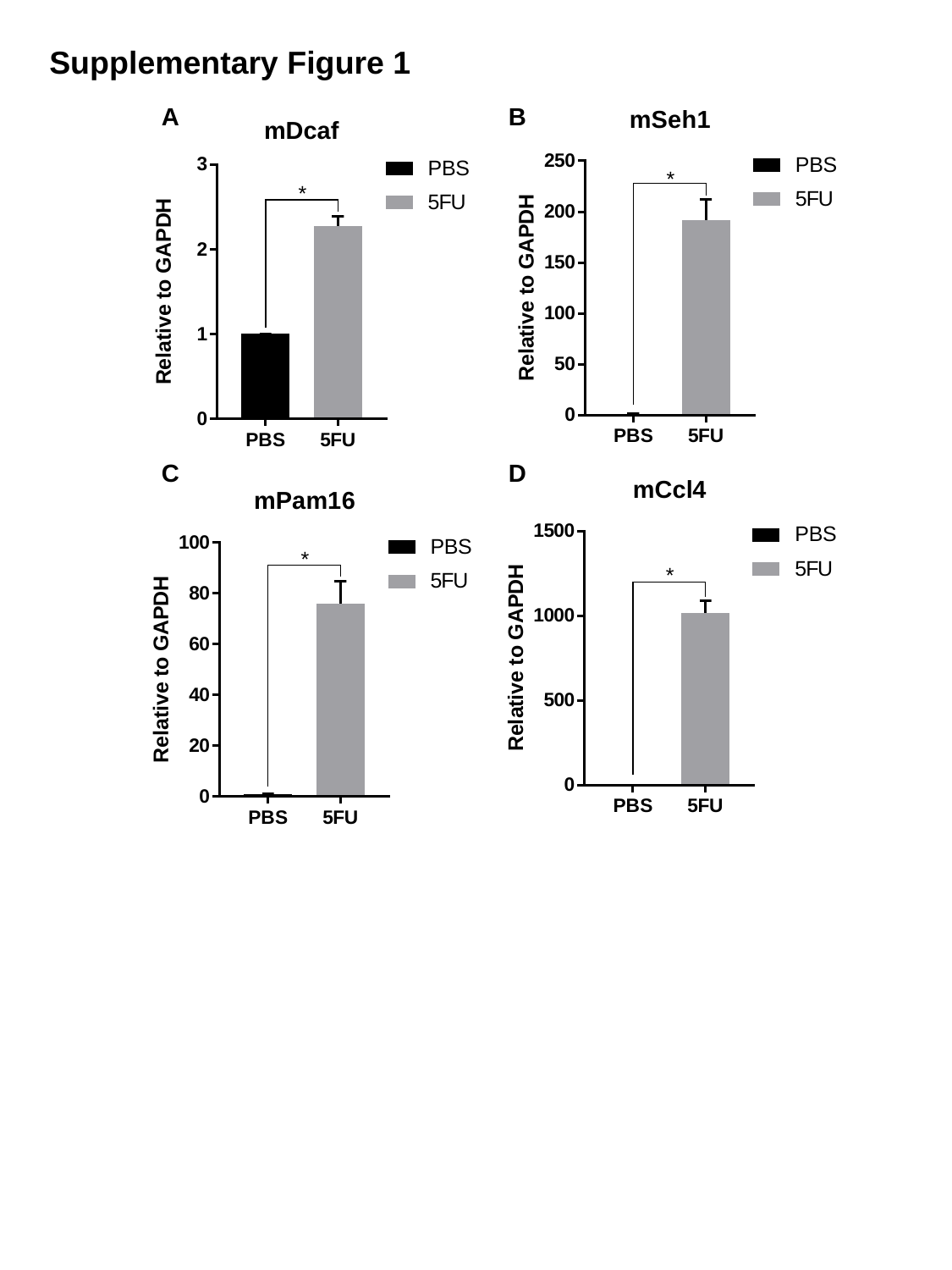

Supplementary Figure 1
A
B
C
D

## Slide 2
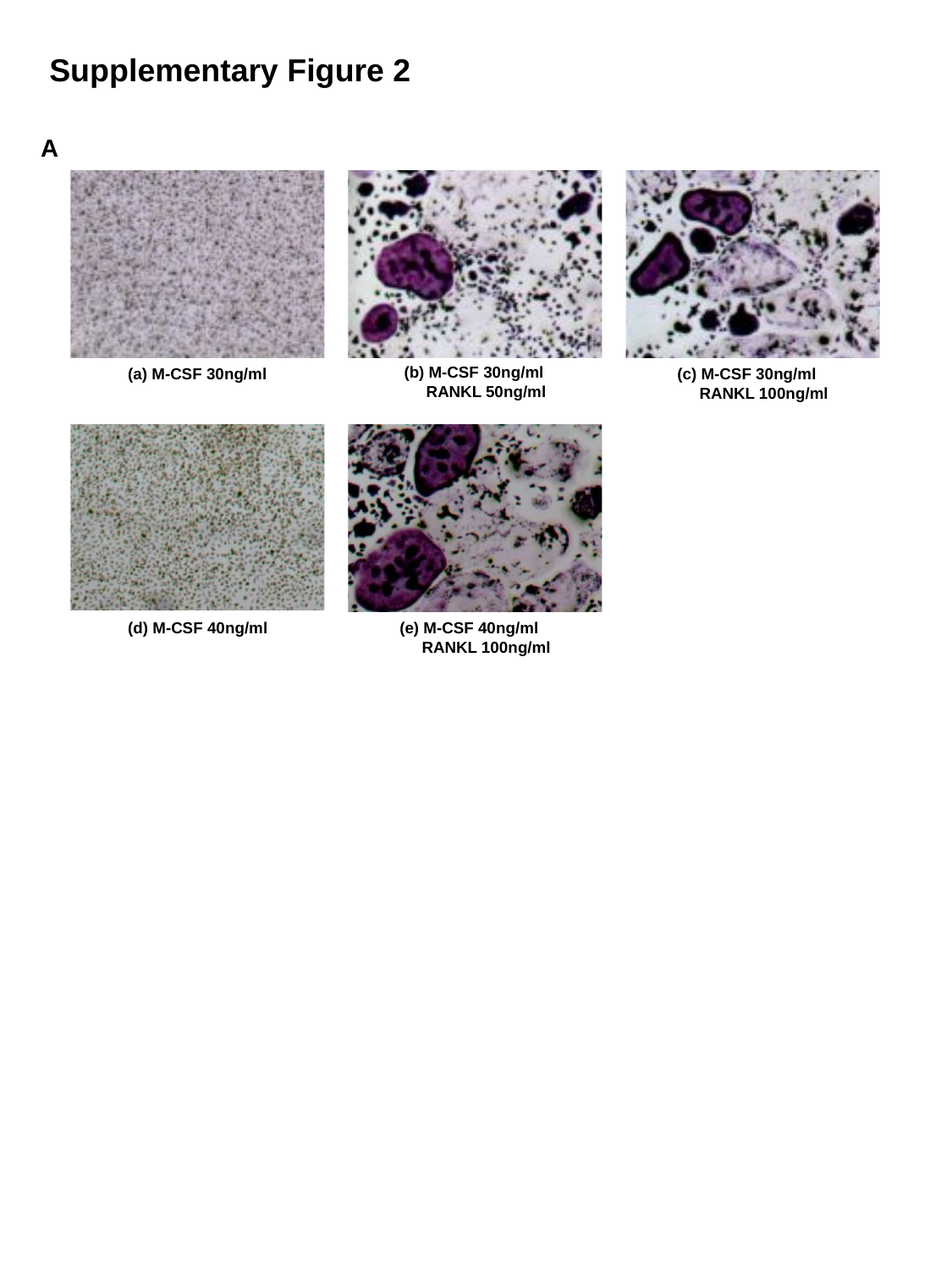

Supplementary Figure 2
A
(b) M-CSF 30ng/ml
 RANKL 50ng/ml
(a) M-CSF 30ng/ml
(c) M-CSF 30ng/ml
 RANKL 100ng/ml
(d) M-CSF 40ng/ml
(e) M-CSF 40ng/ml
 RANKL 100ng/ml

## Slide 3
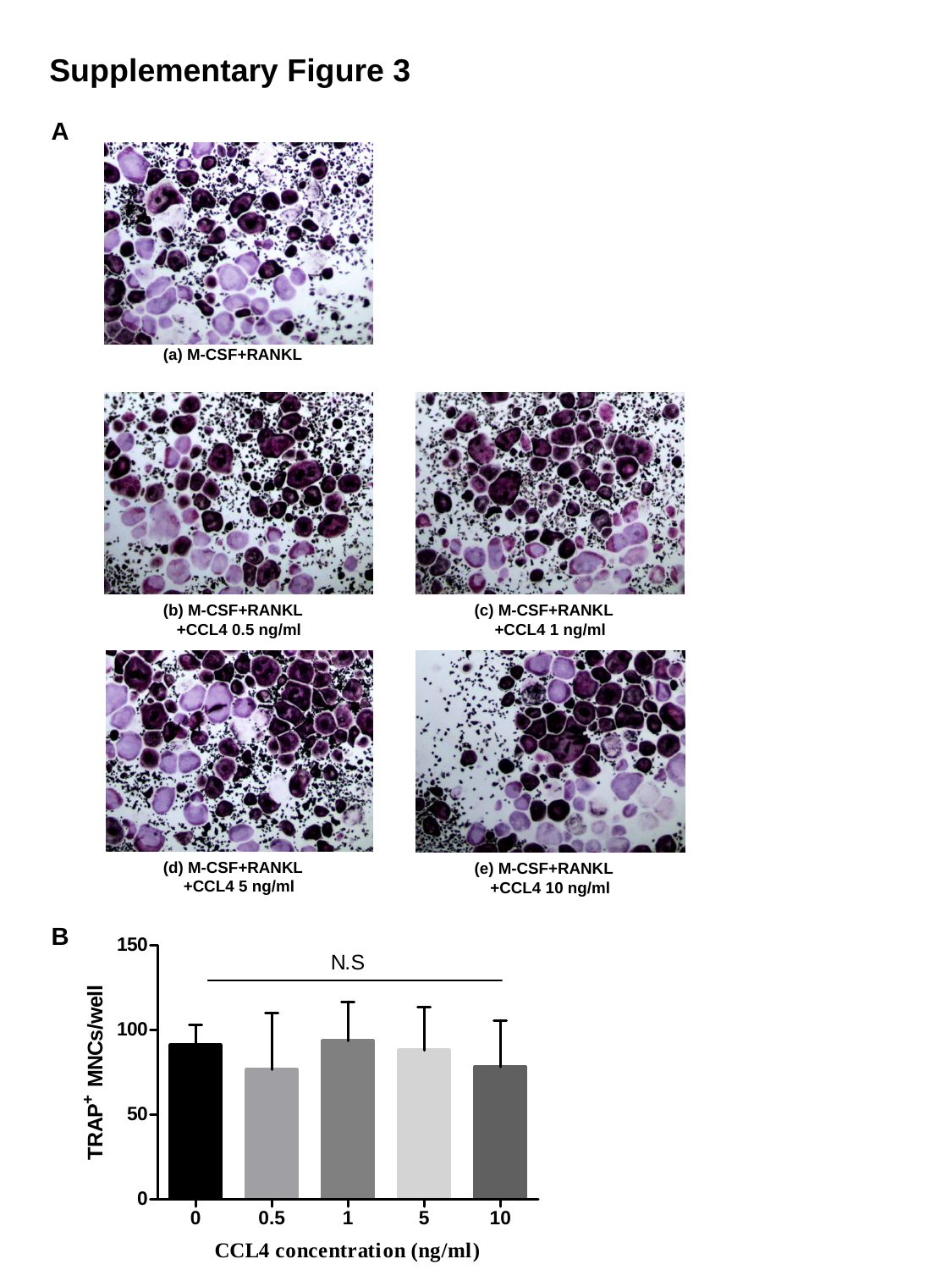

Supplementary Figure 3
A
(a) M-CSF+RANKL
(b) M-CSF+RANKL
+CCL4 0.5 ng/ml
(c) M-CSF+RANKL
+CCL4 1 ng/ml
(d) M-CSF+RANKL
+CCL4 5 ng/ml
(e) M-CSF+RANKL
+CCL4 10 ng/ml
B

## Slide 4
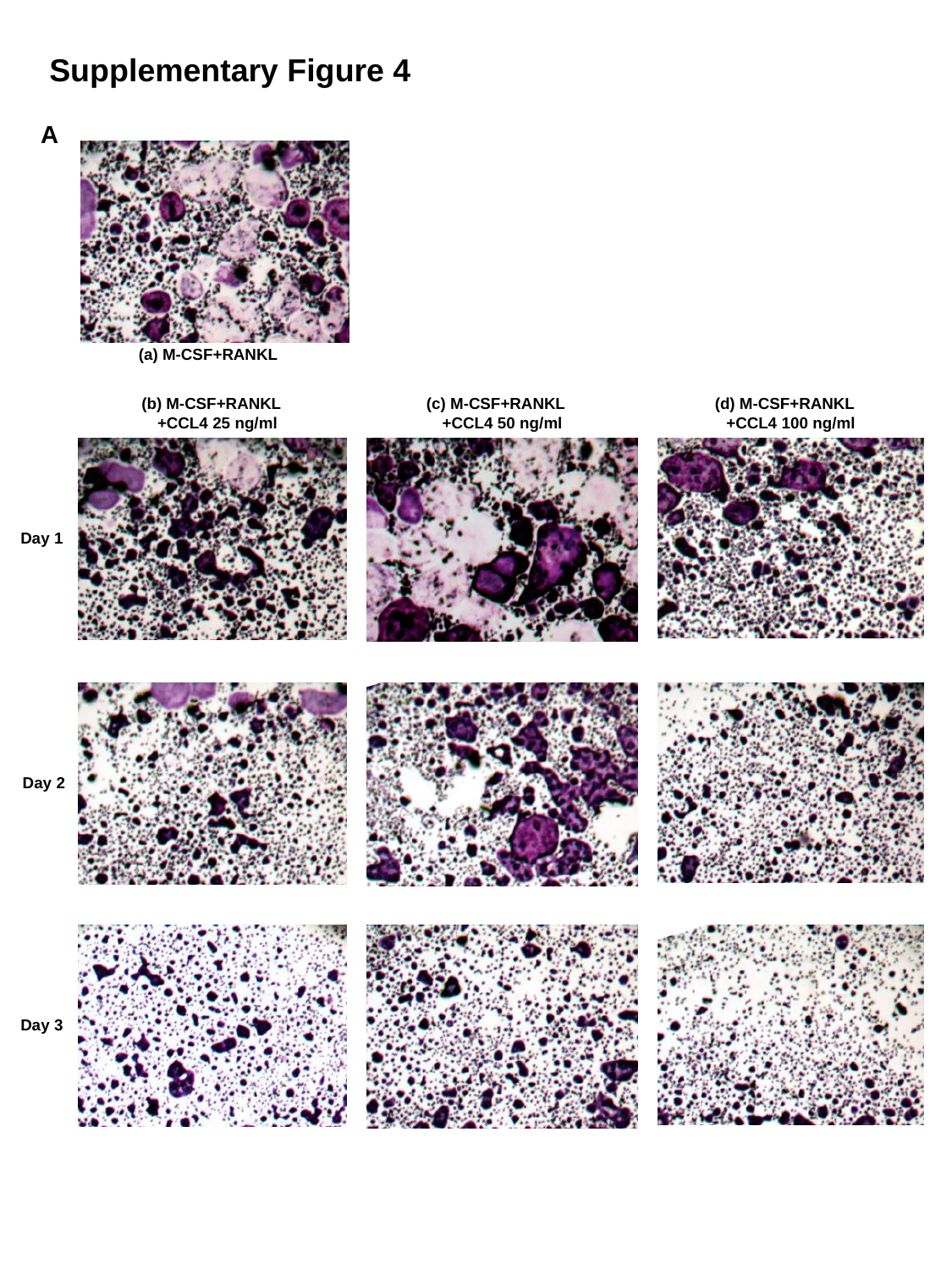

Supplementary Figure 4
A
(a) M-CSF+RANKL
(b) M-CSF+RANKL
+CCL4 25 ng/ml
(c) M-CSF+RANKL
+CCL4 50 ng/ml
(d) M-CSF+RANKL
+CCL4 100 ng/ml
Day 1
Day 2
Day 3

## Slide 5
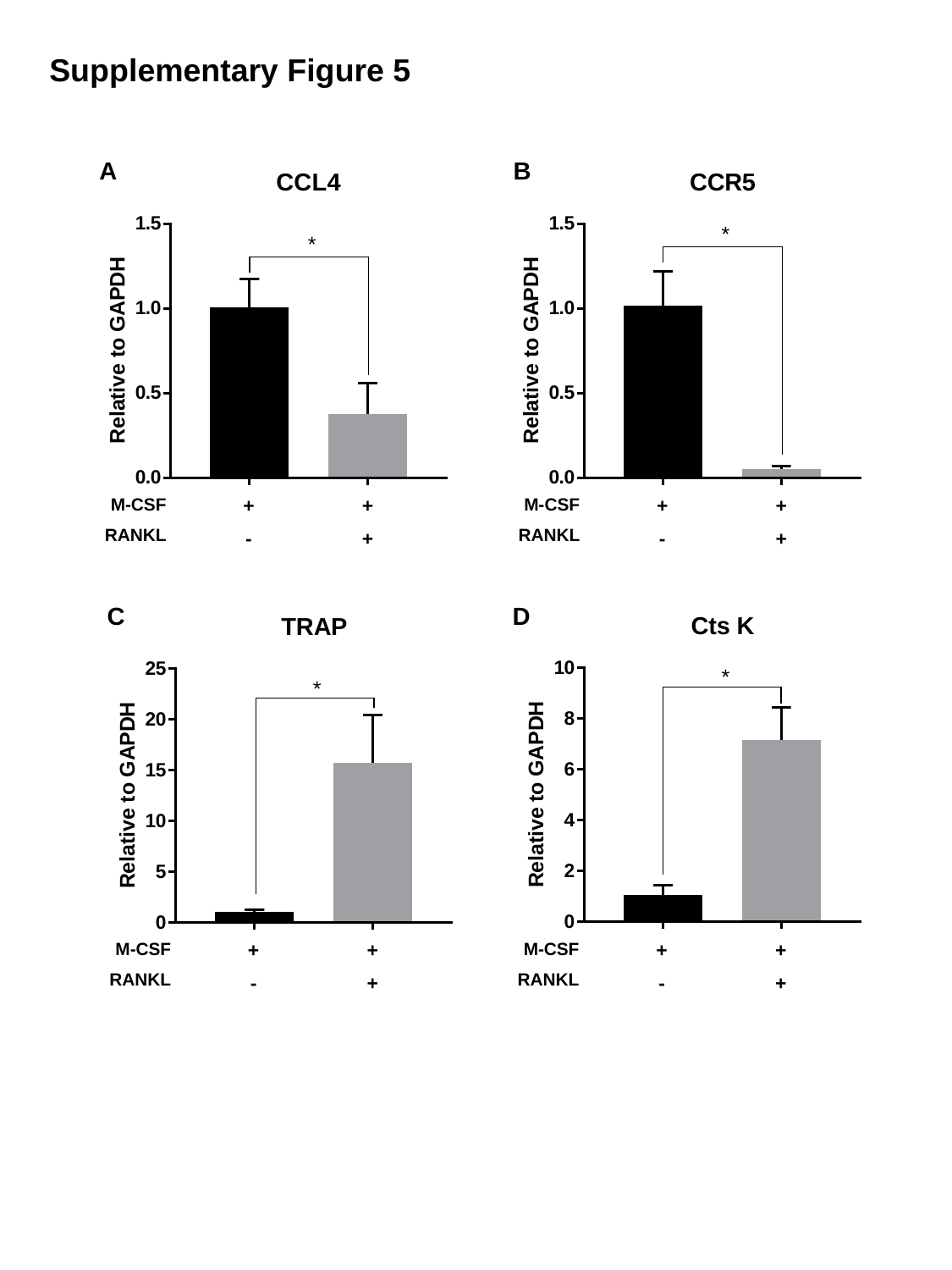

Supplementary Figure 5
A
B
| M-CSF |
| --- |
| RANKL |
| + | + |
| --- | --- |
| - | + |
| M-CSF |
| --- |
| RANKL |
| + | + |
| --- | --- |
| - | + |
C
D
| M-CSF |
| --- |
| RANKL |
| + | + |
| --- | --- |
| - | + |
| M-CSF |
| --- |
| RANKL |
| + | + |
| --- | --- |
| - | + |

## Slide 6
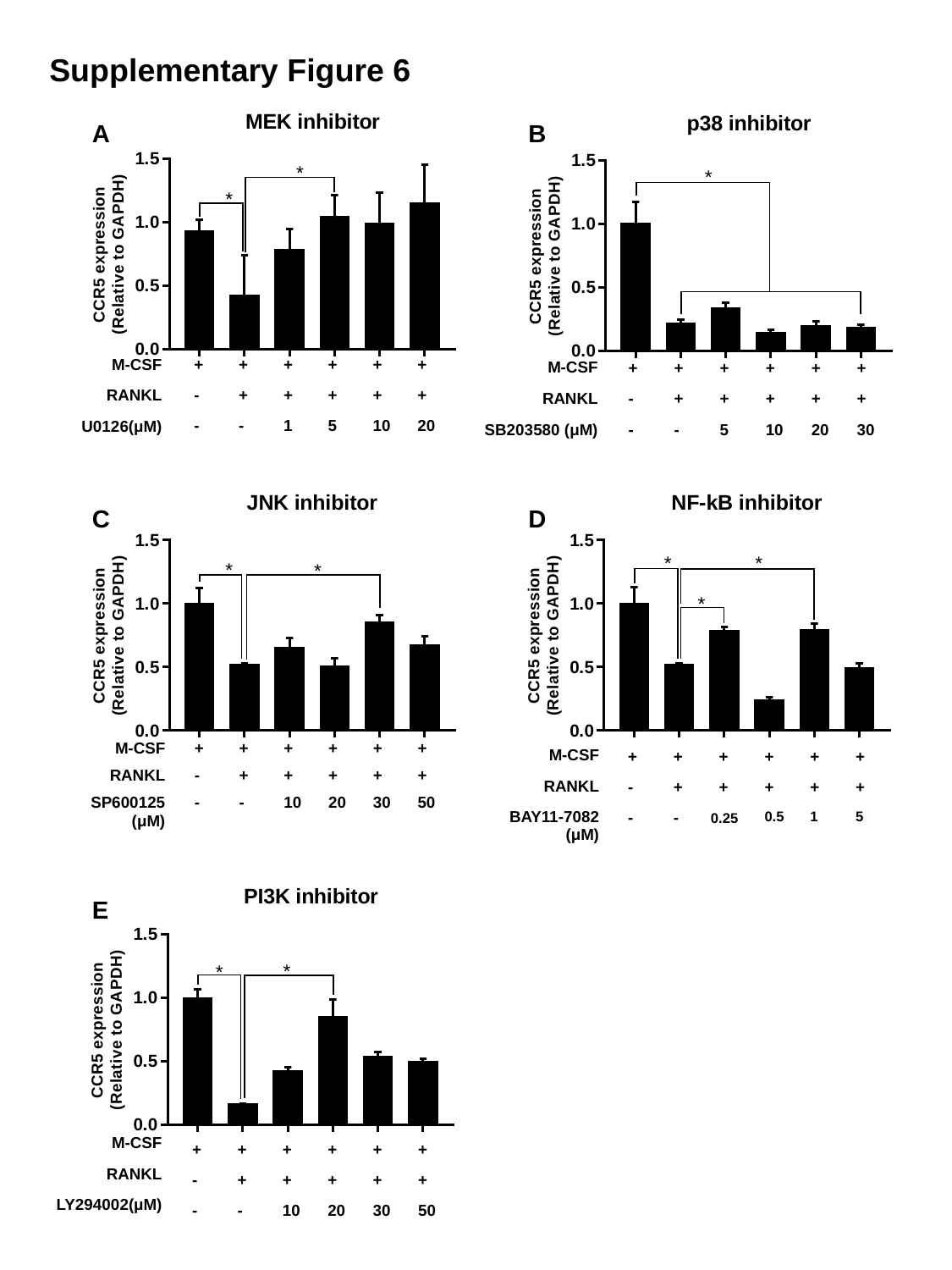

Supplementary Figure 6
A
B
| M-CSF |
| --- |
| RANKL |
| U0126(μM) |
| + | + | + | + | + | + |
| --- | --- | --- | --- | --- | --- |
| - | + | + | + | + | + |
| - | - | 1 | 5 | 10 | 20 |
| M-CSF |
| --- |
| RANKL |
| SB203580 (μM) |
| + | + | + | + | + | + |
| --- | --- | --- | --- | --- | --- |
| - | + | + | + | + | + |
| - | - | 5 | 10 | 20 | 30 |
C
D
| M-CSF |
| --- |
| RANKL |
| SP600125 (μM) |
| + | + | + | + | + | + |
| --- | --- | --- | --- | --- | --- |
| - | + | + | + | + | + |
| - | - | 10 | 20 | 30 | 50 |
| M-CSF |
| --- |
| RANKL |
| BAY11-7082 (μM) |
| + | + | + | + | + | + |
| --- | --- | --- | --- | --- | --- |
| - | + | + | + | + | + |
| - | - 0.25 | | 0.5 | 1 | 5 |
E
| M-CSF |
| --- |
| RANKL |
| LY294002(μM) |
| + | + | + | + | + | + |
| --- | --- | --- | --- | --- | --- |
| - | + | + | + | + | + |
| - | - | 10 | 20 | 30 | 50 |

## Slide 7
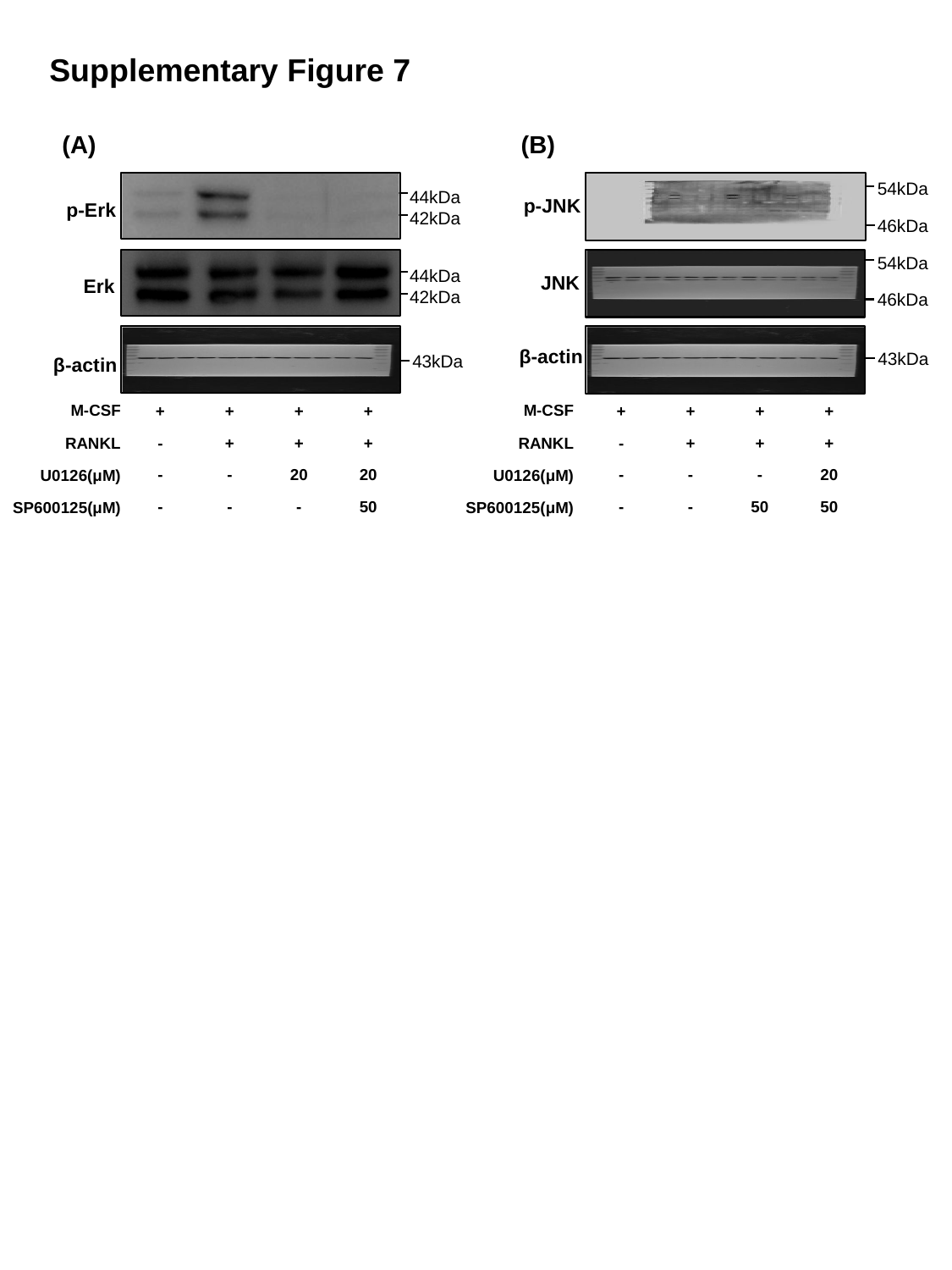

Supplementary Figure 7
(A)
(B)
54kDa
p-JNK
46kDa
54kDa
JNK
46kDa
β-actin
43kDa
44kDa
42kDa
p-Erk
44kDa
42kDa
Erk
43kDa
β-actin
| + | + | + | + |
| --- | --- | --- | --- |
| - | + | + | + |
| - | - | 20 | 20 |
| - | - | - | 50 |
| + | + | + | + |
| --- | --- | --- | --- |
| - | + | + | + |
| - | - | - | 20 |
| - | - | 50 | 50 |
| M-CSF |
| --- |
| RANKL |
| U0126(μM) |
| SP600125(μM) |
| M-CSF |
| --- |
| RANKL |
| U0126(μM) |
| SP600125(μM) |

## Slide 8
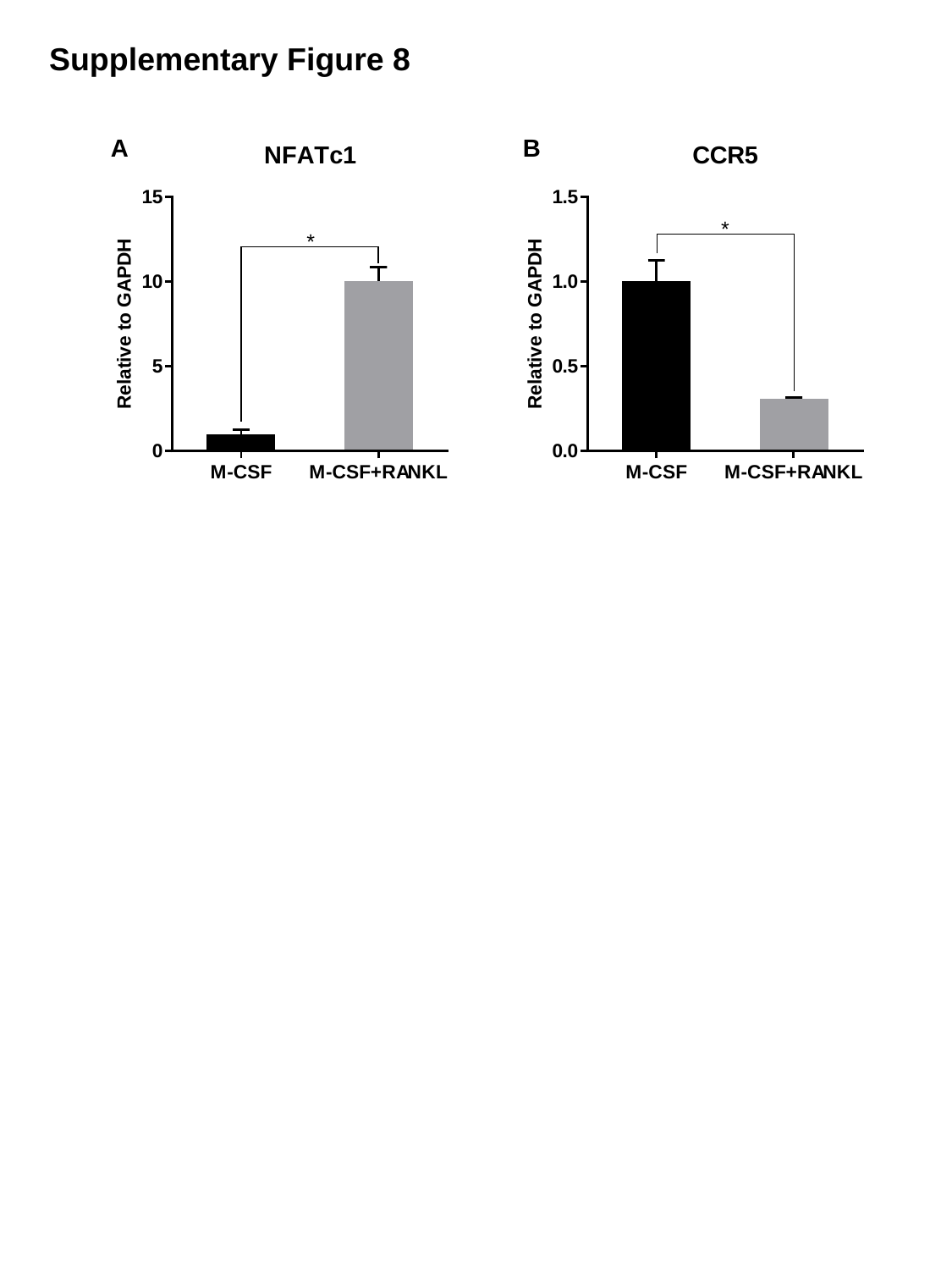

Supplementary Figure 8
A
B
