# Supplementary material for: CCL4 enhances preosteoclast migration and its receptor CCR5 downregulation by RANKL promotes osteoclastogenesis
Source: Cell Death Dis. 2018 May 2;9(5):495. doi: 10.1038/s41419-018-0562-5 (PMC5931580; doi:10.1038/s41419-018-0562-5)
Supplement: Supplementary file 3 — Supplementary figure legends [file 41419_2018_562_MOESM3_ESM.docx]

**Supplementary legends**

**Supplementary Table 1. Up-regulated genes in Osx-positive osteoprogenitor cells by RNA-seq after 5FU treatment.**

**Supplementary Figure 1. Validation of expression level of candidate genes from the RNA-seq analysis in 5FU-activated Osx-positive cells.**

**(A)** Expression of mDcaf; **(B)** expression of mPam16; **(C)** expression of mSeh1; **(D)** expression of CCL4. The data are presented as the mean ± S.D. (n=3; *P-value < 0.05, Student’s t-test)

**Supplementary Figure 2. Effect concentration of M-CSF and RANKL-induced osteoclast differentiation.**

**(A)** The effect of M-CSF and RANKL on osteoclast differentiation was examined with concentration of (a) M-CSF (30 ng/ml), (b) M-CSF (30 ng/ml) + RANKL (50 ng/ml), (c) M-CSF (30 ng/ml) + RANKL (100ng/ml), (d) M-CSF (40 ng/ml) or (e) M-CSF (40ng/ml) + RANKL (100 ng/ml).

**Supplementary Figure 3. CCL4 treatment has no effect on osteoclast differentiation.**

**(A)** The effect of CCL4 on osteoclast differentiation was examined with concentration of (a) 0ng/ml, (b) 500 pg/ml, (c) 1 ng/ml, (d) 5 ng/ml and (e) 10 ng/ml. **(B)** TRAP-positive cells were counted as osteoclasts (≥3 nuclei).

**Supplementary Figure 4. High concentration of CCL4 treatment induced cell death.**

High concentration of CCL4 (b) 25 ng/ml, (c) 50 ng/ml, and (d) 100 ng/ml was treated into cells and differentiated for 3 days

**Supplementary Figure 5. CCL4 is not induced in osteoclast differentiation.**

The expression level of **(A)** CCL4, **(B)** CCR5, **(C)** TRAP and **(D)** Cathepsin K mRNA during osteoclast differentiation. mRNA levels were measured by real-time RT-PCR and the data are presented as the mean ± S.D. (n=3; * P-value < 0.05, One-way ANOVA)

**Supplementary Figure 6. Inhibition of the RANKL-induced reduction of CCR5 mRNA by several inhibitors.**

Pre-osteoclast cells were treated with or without **(A)** U0126, **(B)** SB203580, **(C)** SP600125, **(D)** BAY11-7082 and **(E)** LY294002 at different concentrations for 30 min, followed by RANKL (50 ng/ml) for 24 hr. Expression levels of CCR5 mRNA were measured by real-time RT-PCR and normalized to GAPDH. The data are presented as the mean ± S.D. (n=3; * P-value < 0.05 One-way ANOVA) All the chemicals were dissolved in DMSO and used as a vehicle control in all experiments.

**Supplementary Figure 7. Inhibition of MEK and JNK at the protein activation by MEK and JNK inhibitor**

MEK inhibitor and JNK inhibitor were treated at U0126 (20 μM), SP600125 (50 μM) and U0126 (20 μM) + SP600125 (50 μM) before stimulation with RANKL (50ng/ml) for 15min. **(A)** Phospho-MEK, **(B)** phospho-JNK expression were determined by western blot. All the chemicals were dissolved in DMSO and used as a vehicle control in all experiments.

**Supplementary Figure 8. mRNA level of NFATc1 and CCR5 when treated with RANKL.** The expression levels of **(A)** NFATc1 and **(B)** CCR5 mRNA in pre-osteoclast cells with M-CSF and RANKL. mRNA levels were measured by real-time RT-PCR and the data are presented as the mean ± S.D. (n=3; * P-value < 0.05, One-way ANOVA)
